# Hidden Markov movement models reveal diverse seasonal movement patterns in two North American ungulates

**DOI:** 10.1002/ece3.10282

**Published:** 2023-07-20

**Authors:** John Terrill Paterson, Aaron N. Johnston, Anna C. Ortega, Cody Wallace, Matthew Kauffman

**Affiliations:** ^1^ U.S. Geological Survey Northern Rocky Mountain Science Center Bozeman Montana USA; ^2^ Wyoming Cooperative Fish and Wildlife Research Unit, Department of Zoology and Physiology University of Wyoming Laramie Wyoming USA; ^3^ U.S. Geological Survey, Wyoming Cooperative Fish and Wildlife Research Unit, Department of Zoology and Physiology University of Wyoming Laramie Wyoming USA

**Keywords:** Hidden Markov movement model, migration, movement, mule deer, pronghorn

## Abstract

Animal movement is the mechanism connecting landscapes to fitness, and understanding variation in seasonal animal movements has benefited from the analysis and categorization of animal displacement. However, seasonal movement patterns can defy classification when movements are highly variable. Hidden Markov movement models (HMMs) are a class of latent‐state models well‐suited to modeling movement data. Here, we used HMMs to assess seasonal patterns of variation in the movement of pronghorn (*Antilocapra americana*), a species known for variable seasonal movements that challenge analytical approaches, while using a population of mule deer (*Odocoileus hemionus*), for whom seasonal movements are well‐documented, as a comparison. We used population‐level HMMs in a Bayesian framework to estimate a seasonal trend in the daily probability of transitioning between a short‐distance local movement state and a long‐distance movement state. The estimated seasonal patterns of movements in mule deer closely aligned with prior work based on indices of animal displacement: a short period of long‐distance movements in the fall season and again in the spring, consistent with migrations to and from seasonal ranges. We found seasonal movement patterns for pronghorn were more variable, as a period of long‐distance movements in the fall was followed by a winter period in which pronghorn were much more likely to further initiate and remain in a long‐distance movement pattern compared with the movement patterns of mule deer. Overall, pronghorn were simply more likely to be in a long‐distance movement pattern throughout the year. Hidden Markov movement models provide inference on seasonal movements similar to other methods, while providing a robust framework to understand movement patterns on shorter timescales and for more challenging movement patterns. Hidden Markov movement models can allow a rigorous assessment of the drivers of changes in movement patterns such as extreme weather events and land development, important for management and conservation.

## Graphical abstract

Hidden Markov movement models are well‐suited to understanding seasonal movement patterns. We highlight the differences in seasonal movements between mule deer and pronghorn.

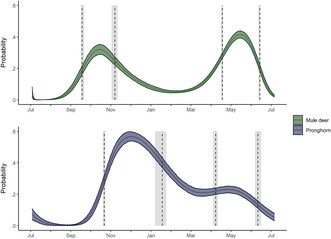

## 1 INTRODUCTION

Animals use different strategies to cope with seasonal differences in resource availability, predation, and climatic conditions (Kie, 1999; Roff, 1993). Animals are constrained in how they allocate energy resources to survival and reproduction, and maximizing individual fitness requires individuals to respond to seasonal changes to optimize energy acquisition and allocation (Promislow & Harvey, 1990). Animal movement is the key mechanism that mediates the connection between a varying environment and fitness, as animals respond to environmental pressures through varying patterns of space use to take advantage of high‐quality nutrition, and to avoid predators and/or environmental extremes (Alerstam et al., 2003; Laskowski et al., 2021; Stamps, 2007). An emergent property of animal behavior is a diversity of animal movement patterns (Teitelbaum & Mueller, 2019), from diel variation in foraging and resting sites to seasonal long‐distance movements (Fryxell & Sinclair, 1988; Nathan et al., 2008; Owen‐Smith et al., 2010).

General concern over the conservation of ungulate populations in the face of habitat fragmentation, loss of movement corridors, and climate change has led to improved understanding of the seasonal movement patterns of ungulates, as the application of statistical and technical advances to movement data has allowed a better understanding of conservation priorities for ungulates (Apollonio et al., 2017; Kauffman et al., 2021; Middleton et al., 2020). The large‐scale migration of ungulate populations between distinct seasonal ranges represents a spectacular example of ungulate movement to moderate seasonal differences in environmental drivers. The environmental and physiological context can be helpful for determining optimal movement strategies for ungulates (Mueller et al., 2011), and the documentation of partially migratory ungulate populations (populations comprised of residents and migrants) and plasticity in migratory behaviors within individuals (individuals who are resident in 1 year and migrants the next) has improved our understanding by suggesting that large‐scale migration may be only one potential optimization strategy (Chapman et al., 2011; Eggeman et al., 2016). There is also a broad spectrum of seasonal movement behaviors from fully resident to long‐distance migrants, including nomads, dispersers, altitudinal migrants, mixed migrants (Kauffman et al., 2021), as well as disruptions to seasonal range fidelity caused by stochastic environmental factors (Jakes et al., 2018).

The continuum of behaviors that define the spectrum of ungulate movement requires a flexible set of analytical tools to understand the drivers of variation in movement. The analysis of spatial displacement metrics (e.g., net‐squared displacement or NSD) is one such technique that has yielded considerable insight into seasonal movement patterns of animals from multiple taxa (Bunnefeld et al., 2011; Kauffman et al., 2021). Based on the cumulative squared distance from a seasonal starting point, NSD analysis yields a model‐based classification of individual movement behaviors into a series of defined categories (e.g., migration, nomadic, dispersal, home range, or some combination thereof; Bunnefeld et al., 2011). However, model‐based classification of movement behaviors has proved analytically challenging for animals with more complicated patterns of seasonal space use. Issues include the lack of clear seasonal ranges and a propensity for sudden, large‐scale movements from seasonal ranges (Jakes et al., 2018; Singh et al., 2016). Despite these being challenges for models seeking to classify behavior using simple displacement metrics, they present an opportunity to understand variation in seasonal ungulate movements by using models with a more flexible temporal scale.

Hidden Markov movement models (HMMs) are an alternative modeling framework useful for understanding variation in movement patterns across temporal scales. A class of latent state models, HMMs are based on two stochastic processes: a process governing the transitions between a discrete set of latent states (used as proxies for behavioral states), and a movement process conditional on the underlying states (Langrock et al., 2012; Leos‐Barajas et al., 2017). They are a robust framework for modeling time series of animal movements because they account for the serial autocorrelation in observations and explicitly model the state and observation processes, allowing for the detection of sources of variation in state changes (McClintock et al., 2020). Hidden Markov movement models have been used to identify behavioral states at multiple temporal scales in a variety of taxa, including daily behaviors of African lions (*Leo leo*; Goodall et al., 2019) and migration behaviors of white sharks (*Carcharodon carcharias*; Weng et al., 2007). However, to the best of our knowledge, HMMs have been underutilized as a method to understand seasonal variation in the movement patterns of ungulates, likely due to the technical resources required for their estimation. Although HMMs and analyses of NSD generally use the same data (i.e., location information from GPS‐equipped collars), the flexible temporal scale(s) for the state‐transition process definition of the former allows for a finer‐grained classification of seasonal movement behaviors.

Here, we took a comparative approach to assess the utility of using population‐level, two‐state HMMs (interpreted as a short‐distance local movement state, SDLM, and a long‐distance movement, LDM, state) for quantifying seasonal movement patterns of two ungulate species. Mule deer (*Odocoileus hemionus*) are native to western North America, and in southwestern Wyoming are overwhelmingly migratory with some of the longest documented migrations between seasonal ranges of any ungulate in the continental United States (Kauffman et al., 2020). Pronghorn (*Antilocapra americana*) are also native to western North America, and Wyoming represents a large portion of their historical range (O'Gara & Yoakum, 2004). Although less is known about their movement ecology, pronghorn have flexible movement patterns, including frequent facultative movements deviating from winter ranges in response to challenging environmental conditions (Jakes et al., 2018). In this study, we assessed the seasonal patterns of movements and the variations and transitions within these patterns for mule deer and pronghorn. First, we expected that the seasonal patterns of variation in state transitions between short‐distance and long‐distance movement states for mule deer would reflect a strongly seasonal trend consistent with migration in the fall to winter ranges and migration in the spring to summer ranges. Second, we expected that pronghorn should show a seasonal trend consistent with migration to seasonal ranges similar to mule deer; however, their response to extreme winter conditions would result in higher probabilities of long‐distance movements during winter. Finally, we expected overall more variable patterns of switching between movement states for pronghorn compared to mule deer, given the available evidence suggesting they have more flexible and less predictable movements (Jakes et al., 2018; Milligan et al., 2023).

## 2 METHODS

### 2.1 Study areas

**FIGURE 1:**
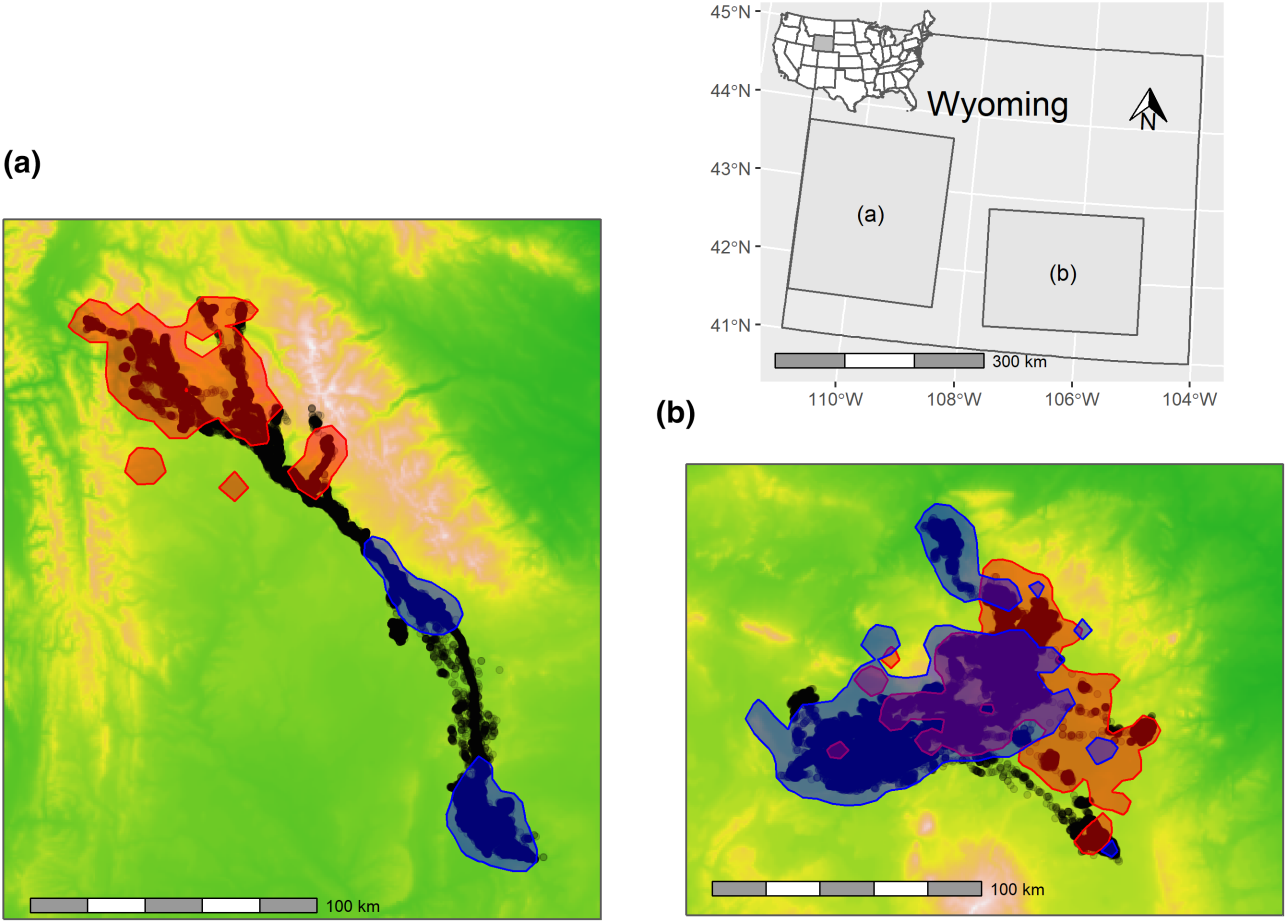
Study areas for mule deer (a) and pronghorn (b) in Wyoming, USA. A random sample of animal locations (black dots) from 25 animals (for both mule deer, 197,824 locations, and pronghorn, 126,492 locations) indicates general space use across years, and the 95% isopleth of kernel density estimates for summer locations (generically defined as June–August, red), and winter locations (generically defined as December–February, blue) suggest broad seasonal space use patterns. The background colors denote elevation and range from approximately 1500–3900 m for mule deer, and 1400–3500 m for pronghorn.

We used GPS collar data from adult female pronghorn (>1‐year‐old) in the Medicine Bow Herd that spends the winter in the Shirley Basin of central Wyoming and from adult female mule deer (>1‐year‐old) in a portion of the Sublette Herd that spends the winter in the Red Desert of southcentral Wyoming, USA (Figure 1). The pronghorn study area was a sagebrush‐steppe dominated by Wyoming big sagebrush (*Artemesia tridentata*). Elevations ranged from 1366 to 3597 m. For mule deer, the long‐distance migratory individuals in the Red Desert population of migrate >130 km one‐way to the summer ranges in the Hoback and upper Green River Basins. Summer ranges for these long‐distance migrants are characterized as montane and subalpine forests and are dominated by lodgepole pine, subalpine fir, aspen, western snowberry, lupine, yarrow, and sticky purple sweetvetch. Winter ranges in the Red Desert are characterized as a desert and sagebrush shrubland dominated by big sagebrush, antelope bitterbrush, rabbitbrush, greasewood, and native bunchgrasses, including needle‐and‐thread. Elevations across the study area ranged from 1479 to 4929 m.

### 2.2 Captures

In March 2018, we captured adult female pronghorn (*n* = 80) in the Shirley Basin, near Medicine Bow, Wyoming, USA, and replaced collars each March and November/December to maintain a consistent sample size of adult female pronghorn (Supporting Information). Pronghorn were captured by helicopter net gunning and outfitted with store‐on‐board GPS collars that collected locations every 2 h and transmitted one location remotely a day (model RECON‐4560‐4; Telonics). Animal capture and handling protocols for pronghorn were approved by Wyoming Game and Fish Department (Chapter 33–1162) and University of Wyoming Institutional Animal Care and Use Committee (protocol 20180306MK00297‐03).

From 2014 to 2021, we captured *n* = 253 adult female mule deer (>1‐year‐old) in the Red Desert via helicopter net‐gunning. We outfitted all deer with store‐on‐board or iridium GPS collars that collected locations every 1–2 h (Advanced Telemetry Systems, Inc; LOTEK Wireless Inc; Telonics Inc). All animal capture and handling protocols were approved by the Wyoming Game and Fish Department (Chapter 33–937) and an Institutional Animal Care and Use Committee at the University of Wyoming (Protocol 20131111KM00040, 20151204KM00135, 20170215KM00260, 20200302MK00411).

### 2.3 Data processing

We first defined a biological year from July 1 to June 30 in order to define a year as the period beginning and ending when most animals were likely to begin on summer range and treated each animal‐year (the location data for each animal within a biological year) as an independent time series of locations. Within each animal‐year, we resampled the location information to one location per day (the point closest in time to 6 a.m.) to focus on daily variation in movement characteristics within the year. We used the amt package (Signer et al., 2019) in the R programming environment (R Core Team, 2020) to process the results by calculating the step length in kilometers between daily locations, and the turning angle as the deviation (in radians) from the bearing defined by the previous two daily locations. Missing data in the GPS signal occasionally resulted in successive locations separated by more than a day (approximately 8% of total locations for mule deer, <1% for pronghorn). To prevent bias in estimated movement characteristics induced by these gaps, we treated the step length and turning angle for the missing location as missing data to model the complete time series of movement data.

### 2.4 Hidden Markov movement model

We used a 2‐state HMM (state 1 = short‐distance local movement, SDLM; state 2 = long‐distance movement, LDM) to model daily animal movements, where the unobserved behavioral state was estimated from the daily step lengths (km) and turning angles (radians; Leos‐Barajas et al., 2017; McClintock et al., 2020). Separate models were fit for pronghorn and mule deer: each model was time‐invariant at the population level with each animal‐year treated as an independent time series (i.e., observations across years and individuals were completely pooled). We restricted our analysis to a single 2‐state model per species (i.e., no model comparison to assess evidence for more or less states) to allow for short‐distance local movements (e.g., on annual ranges) to be coupled to a longer‐distance movement state (e.g., migration between ranges), reflecting the strongly seasonal migration patterns of these mule deer (Sawyer et al., 2016). Our goal was to understand seasonal variation in movement characteristics; therefore, we defined a time‐inhomogeneous Markov process where the transition probability matrix ($\Gamma^{t}$) varied as a function of the day of the year, *t*. The state transition probabilities from day *t*−1 (row, *i*) to day *t* (column, *j*), $\gamma_{i,j}^{t}$, were defined using a 2×2 matrix:

(1) $$\;\Gamma^{t}=\left[\begin{array}{cc} {1-\gamma }_{1,2}^{t} & \gamma_{1,2}^{t}\\ \gamma_{2,1}^{t} & {1-\gamma }_{2,1}^{t} \end{array}\right].$$

The daily state transition probabilities ($\gamma_{1,2}^{t}$, $\gamma_{2,1}^{t}$) were modeled using the logit link with a state‐specific intercept ($\beta_{i,j}^{0}$) and a seasonal trend using the day of the biological year (July 1 = 1) as a covariate ($f_{i,j}\left(\mathrm{day}\;\text{of the year}\right)$):

(2) $$\text{logit}\left(\gamma_{i,j}^{t}\right)=\beta_{i,j}^{0}+f_{i,j}\left(\mathrm{day}\;\text{of the year}\right),i\neq j.$$

We used a spline formulation for the seasonal trend, $f_{i,j}\left(\text{day of the year}\right)$, using an a priori definition of 5 knots evenly spaced from 1 to 365, which initial modeling efforts suggested was an adequate number to capture seasonal trends and a compromise for estimation efficiency (Supporting Information). We adopted a Bayesian interpretation of splines that used a random‐effects structure to relate the spline basis Z to the transition probability from state *i* to *j* using regression coefficients (b):

(3) $$f_{i,j}\left(\mathrm{day}\;\text{of the year}\right)=\sum_{k=1}^{5}b_{i,j}^{k}Z_{\mathrm{day}\;\text{of the year},k},$$

where $b_{i,j}^{k}\sim \text{Normal}\left(0,\sigma_{i}\right),i\neq j$ (Crainiceanu et al., 2005). We modeled state‐specific step lengths and turning angles between daily locations using a gamma distribution and a wrapped Cauchy distribution, respectively. For each state (*i =* 1, 2):

(4) $$\text{step length}\;|\;\text{state}=i\sim \text{Gamma}\left(\alpha_{i},\beta_{i}\right),$$

(5) $$\text{turning angle}\;|\;\text{state}=i\sim \text{wrapped Cauchy}\left(\mu_{i},\kappa_{i}\right),$$

where $\alpha_{i}$ and $\beta_{i}$ correspond to the shape and rate parameters of a Gamma distribution, and $\mu_{i}$ and $\kappa_{i}$ correspond to the mean direction (in radians) and concentration of a wrapped Cauchy distribution.

### 2.5 Model estimation and details

We used a Bayesian framework to take advantage of the ability to estimate derived parameters, and flexible model statements available from the nimble package (de Valpine et al., 2022) in the R environment (R Core Team, 2020; Supporting Information). We found that defining priors on the mean ($\mu_{1}^{\text{gamma}}\sim \text{Truncated Normal}\left(0,\sigma =10\right)$, truncated at 0 to be strictly positive) and standard deviation ($\sigma_{i}\sim \text{Truncated Normal}\left(0,\sigma =10\right)$) of the gamma distribution (rather than priors on the shape and rate) improved convergence. To ameliorate the label‐switching problem of Bayesian mixture models (Leos‐Barajas & Michelot, 2018), we modeled the mean of the gamma distribution for the step lengths of the second state as $\mu_{2}^{\text{gamma}}=\mu_{1}^{\text{gamma}}+\delta$, where $\delta \sim \text{Truncated Normal}\left(0,\sigma =10\right)$ to ensure the second state was defined by larger step lengths. For the distributions of turning angles, we assigned diffuse priors to both the state‐specific means ($\mu_{i}\sim \text{Uniform}\left(-\pi ,\pi \right)$) and concentrations ($\kappa_{i}\sim \text{Uniform}$(0, 1)). We assigned independent diffuse priors to the two intercepts ($\beta_{1,2}^{0},\beta_{2,1}^{0}\sim \text{Normal}\left(0,\sigma =2\right)$) for the daily probabilities of transitions between states on the logit scale.

For the spline model of daily transition probabilities, we used a diffuse prior for the standard deviation of the random effects ($\sigma_{i}\sim \text{Truncated Normal}\left(0,\sigma =10\right)$). The final component of the model was a prior on the initial movement state in each animal record. Our interest was modeling daily probabilities of state transitions; therefore, we modeled the probabilities of each initial movement state for each unique day that began an animal record (*t*) using a $\text{Categorical}\left(\phi^{t}\right)$ prior, where $\phi^{t}\sim \text{Dirichlet}\left(1,1\right)$.

For each species‐specific model, we ran three parallel chains of 20,000 iterations each and discarded the first 10,000 iterations. We thinned the resulting chains by a factor of 5 for memory purposes, resulting in 6000 total samples from which we made inferences. The convergence of the chains was graphically assessed using trace plots and the Gelman–Rubin diagnostic (Gelman & Rubin, 1992) for all the nonderived parameters. We used the median and the 90% highest posterior density interval (HDPI) to summarize the posterior distributions of parameters.

Finally, we performed a post hoc analysis to help clarify the seasonal patterns in transition probabilities using the posterior distributions of transition probabilities and time‐specific initial state distributions to estimate the marginal probabilities of being in the LDM state on every day of the year (i.e., the probability of being in the LDM state on day *t* regardless of behavioral state on day *t*−1) (Figure 4). We then defined seasonal events in this probability using the first derivative (fall start = first maximum, fall end = first minimum, spring start = second maximum, and spring end = second minimum) and derived estimates of the timing of these events.

## 3 RESULTS

The pronghorn collaring effort resulted in 778,788 locations from 150 animals over four calendar years (2018–2021), for a total of 345 animal‐years of location information. The mule deer collaring effort resulted in 654,918 locations from 89 animals over 8 years (2014–2021), for a total of 317 animal‐years of location information. The fix rates of the collars for both species were approximately 2 h with more variation in fix rates for mule deer than pronghorn (pronghorn: median = 2 h, range from 0.11 to 184 h; mule deer: median = 2 h, range from 0.12 to 1491 h). After resampling locations to one per day and calculating step lengths and turning angles the pronghorn data set consisted of 65,801 steps and 65,441 turning angles, and the mule deer datasets consisted of 66,401 steps and 64,784 turning angles. Daily step lengths varied among pronghorn animal‐years (median = 1.15 km, range from <100 m to 50.50 km) and mule deer animal‐years (median = 0.59 km, range from <100 m to 63.30 km), and turning angles were distributed from −*π* to *π* radians (Supporting Information).

### 3.1 Movement parameters

**FIGURE 2:**
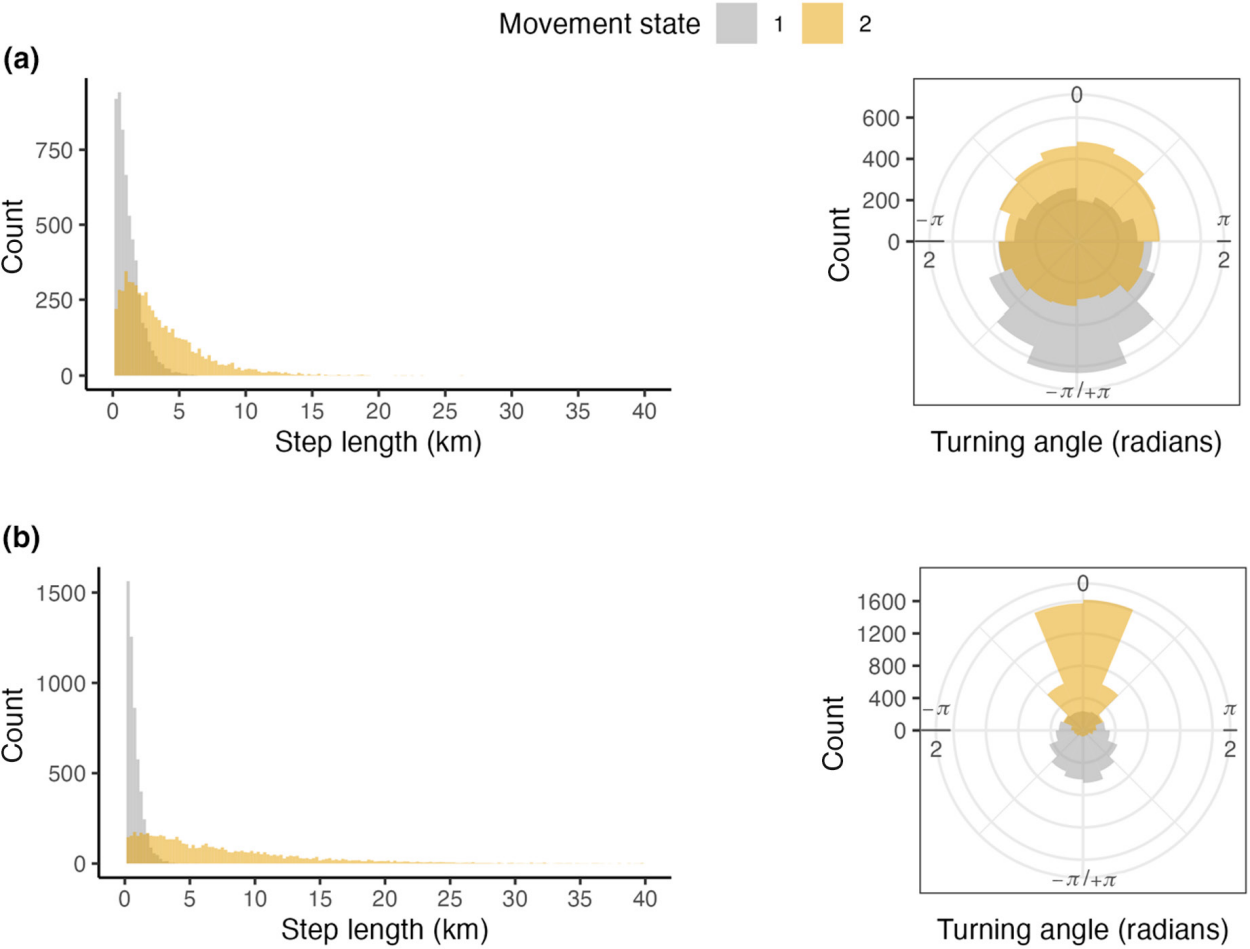
Distributions of movement characteristics (step lengths and turning angles) for each movement state (state 1 equals short‐distance local movements; state 2 equals long‐distance movements) for pronghorn (a) and mule deer (b) from the estimated Hidden Markov movement model. A turning angle equal to 0 radians indicates a straight‐ahead movement path; a turning angle equal to π radians indicates the opposite direction from the previous bearing.

The estimated HMMs for pronghorn and mule deer were broadly similar in that daily movements were separable into two distinctly estimated movement states; however, the parameters of the movement characteristic distributions differed. The estimated parameters of the step length distributions for the SDLM state in pronghorn and mule deer (pronghorn: $\mu_{1}^{\text{gamma}}$ = 1.17 km, 90% HDPI = [1.16, 1.18], $\sigma_{1}$ = 0.947 km [0.95, 0.98]; mule deer: $\mu_{1}^{\text{gamma}}$ = 0.66 km [0.65, 0.66], $\sigma_{1}$ = 0.57 km [0.56, 0.57]) result in similar distributions of random step lengths for pronghorn and mule deer (Figure 2). In contrast, the estimated parameters for the step length distributions for the LDM state (pronghorn: $\mu_{1}^{\text{gamma}}$ = 3.64 km [3.56, 3.72], $\sigma_{1}$ = 3.04 km [2.97, 3.10]; mule deer: $\mu_{1}^{\text{gamma}}$ = 7.12 km [7.01, 7.23], $\sigma_{1}$ = 6.49 km [6.39, 6.60]) resulted in distributions of random step lengths that were generally smaller for pronghorn (Figure 2). Similarly, the estimated parameters of the turning angle distributions for the SDLM state (pronghorn: cos($\mu_{1}$) = −1.00 [−1.00, 1.00], sin($\mu_{1}$) = 0.01 [0.00, 0.02], $\kappa_{1}$ = 0.24 [0.23, 0.25]; mule deer: cos($\mu_{1}$) = −1.00 [−1.00, −1.00], sin($\mu_{1}$) = 0.01 [0.00, 0.02], $\kappa_{1}$ = 0.26 [0.25, 0.26]) result in similar distributions of random turning angles for pronghorn and mule deer (Figure 2). Although the estimated mean of the turning angle distributions for the LDM state for both pronghorn and mule deer were similar (pronghorn: cos($\mu_{2}$) = 1.00 [1.00, 1.00], sin($\mu_{2}$) = −0.01 [−0.03, 0.00]; mule deer: cos($\mu_{2}$) = 1.00 [1.00, 1.00], sin($\mu_{2}$) = 0.02 [0.01, 0.03]), the lower concentration term for pronghorn ($\kappa_{2}$ = 0.12 [0.11, 0.14]) compared to mule deer ($\kappa_{2}$ = 0.70 [0.69, 0.70]) resulted in distributions of random turning angles that were more diffuse for pronghorn (Figure 2).

### 3.2 Seasonality of movement patterns

**FIGURE 3:**
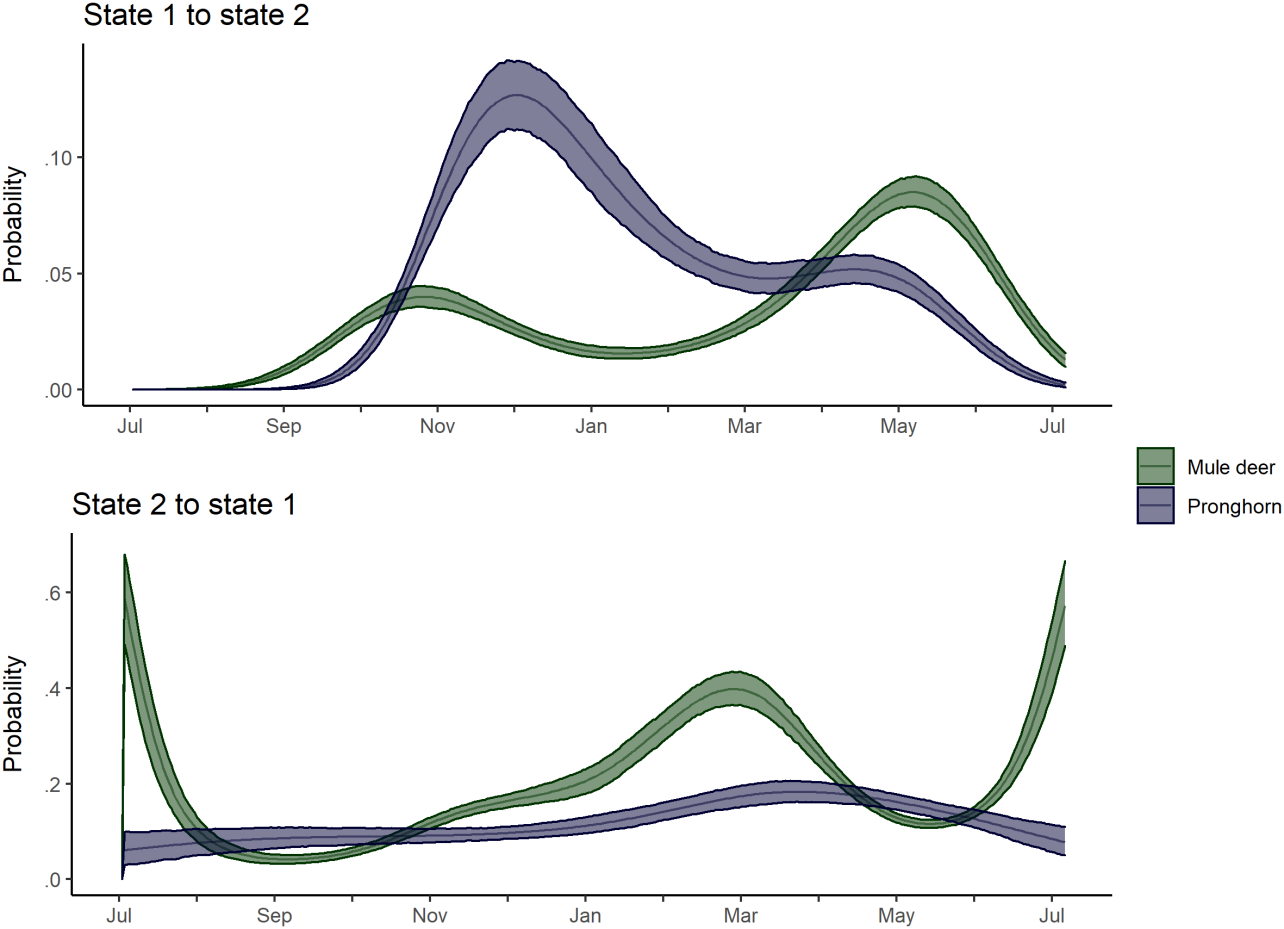
Seasonal trends in the daily probabilities of transitioning among movement states (state 1 equal to short distance local movements; state 2 equal to long distance movements) in the HMM for pronghorn (purple) and mule deer (green). The solid line indicates the median and the ribbon indicates the 90% HDPI. The flexible, spline‐based approach to modeling seasonal movements did not guarantee July–July probabilities be equal, and the minor differences in probabilities arose from the tradeoff between flexibility and simplicity in the model form. Note the differences in scale for the *y*‐axes in both panels.

The estimated daily probabilities of state transitions varied throughout the year for both species, and the seasonal trends were different (Figure 3). The daily probability of transitioning from the SDLM state to the LDM state for pronghorn increased from approximately zero during the summer to a single maximum of 0.13 [0.11, 0.14] on day 151 (December) prior to slowly declining to a minimum of approximately 0 near the end of the biological year (Figure 3, top panel). For mule deer, the daily probabilities had two maxima: The transition probabilities increased from approximately zero during the summer to a local maximum of 0.04 [0.035, 0.045] on Day 114 (late October), declined to a minimum of 0.02 [0.01, 0.02] during the winter (December to February), before again increasing to a global maximum of 0.08 [0.08, 0.09] on Day 306. Although the early seasonal increase in this daily transition probability were similar between the two species, the differences in the daily probabilities of transitioning back to the SDLM state from the LDM state suggested two different trends in movement states (Figure 3, bottom panel). For mule deer, the probability of transitioning back to the SDLM state from the LDM state had a maximum during the summer months (0.59 [0.49, 0.68]), prior to declining to a minimum coincident with the early‐season increase in SDLM to LDM transition probability (0.04 [0.03, 0.05]), an increase over the winter during the winter months (0.40 [0.37, 0.43]), another local minimum coincident with the late‐season increase in the SDLM to LDM state transition probability (0.12 [0.11, 0.12]), and a final increase to a local maximum during the summer months (0.60 [0.51, 0.69]). We interpreted this pattern to suggest that any transition to the LDM state in mule deer in the summer was short‐lived prior to the seasonal increase in the SDLM to LDM state transition in the late fall and was similarly short‐lived during the winter prior to the seasonal increase in the SDLM to LDM state transition in the spring. In contrast, the probabilities of the LDM to SDLM state transition for pronghorn was roughly constant and overall lower than for mule deer (e.g., the probabilities ranged from a minimum of 0.06 [0.03, 0.10] on Day 2 to a probability of 0.18 [0.16, 0.21] on Day 261).

**FIGURE 4:**
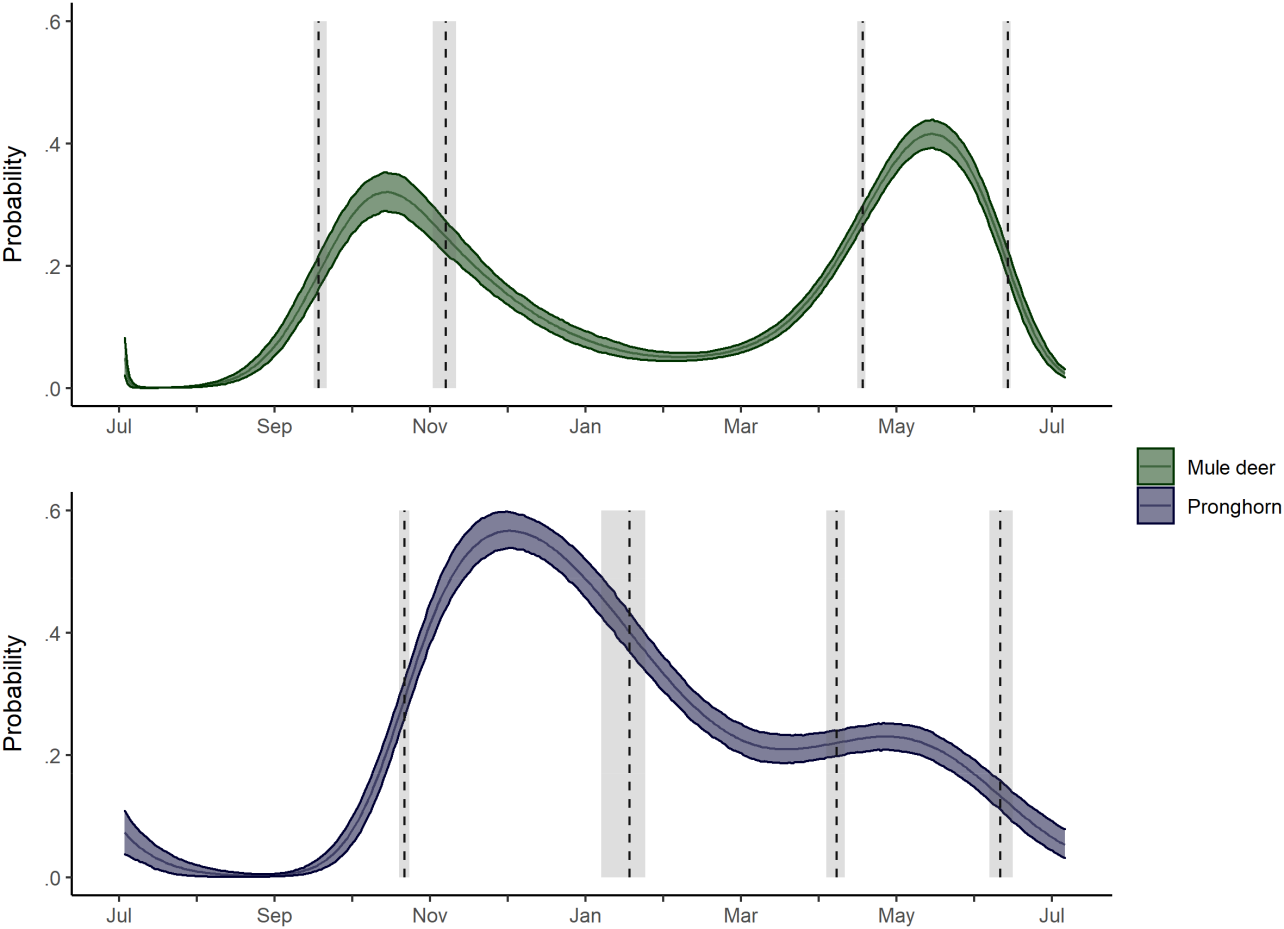
Marginal probabilities of animals being in movement state 2 (long distance movements) on each day of the year for mule deer (green, top panel) and pronghorn (purple, bottom panel). The solid line indicates the median and the ribbon indicates the 90% HDPI. We defined seasonal events as the maximum rate of change in the transition probabilities and estimated the timing of these events using the approximate posterior. The dashed line indicates the median day, and the gray interval indicates the 90% HDPI.

Our post hoc analysis of the marginal probabilities of being in the LDM state revealed strong seasonal differences between the two species (Figure 4). For mule deer, the seasonal patterns in the SDLM to LDM and LDM to SDLM state transitions resulted in a seasonal pattern of being in the LDM state with two local maxima in the fall (0.32 [0.29, 0.35]) and spring (0.42 [0.39, 0.44]) and an overall minimum (0.05 [0.04, 0.06]) during the winter, suggesting two discrete periods with a high probability of LDM movement. This variation translated into a start day for fall movement of day 77 [75, 80], an end day for fall movement of 126 [121, 130], a start day for spring movement of day 287 [285, 288] and an end day for spring movement of 343 [341, 344]. For pronghorn, the seasonal pattern of the probability of being in the LDM state closely resembled the seasonal pattern of the SDLM to LDM transition probability, with a maximum in the late fall of 0.57 [0.54, 0.60], a minimum of 0.21 [0.18, 0.23] in the spring and a maximum of 0.23 [0.21, 0.25] in the late spring. This variation translated into a start day for fall movement of day 110 [108, 112], an end day for fall movement of 197 [186, 203], a start day for spring movement of day 277 [273, 280] and an end day for spring movement of 340 [336, 345].

## 4 DISCUSSION

Hidden Markov movement models are useful tools for understanding sources of variation in animal movement patterns, though they have been underutilized to model seasonal movement patterns in ungulates. We assessed the utility of HMMs for understanding seasonal patterns of variation in movements of mule deer and pronghorn in Wyoming. We found seasonal movement patterns of mule deer were consistent with two discrete periods of long‐distance movement states in the early fall and late spring, coupled to periods of short‐distance local movement states in the summer and winter. Seasonal movement patterns of pronghorn indicated less discrete periods of long‐distance movement states. Although there was an early season increase in the probability of long‐distance movement, this increase was followed by a period of higher probability of long‐distance movement, and a less dramatic transition period to summer ranges in the late spring. We found substantial differences in the timing of seasonal movements between mule deer and pronghorn in the fall and early winter using parameters derived from estimated transition probabilities. In aggregate, we found that HMMs for the analysis of seasonal movement data yielded similar inference to more standard approaches for species with well‐understood movement patterns, while also allowing for a more flexible and robust approach to better understand movements of species with more variable and harder‐to‐define patterns.

Our results largely confirm the general insights based on analyses of NSD for portions of the Red Desert mule deer. These migratory mule deer make expansive northward movements from winter ranges to summer ranges in the Hoback and upper Green River basins (Sawyer et al., 2014, 2016). The start dates of spring migrations have previously been estimated from early April to early May and fall migrations from mid‐ to late October, the exact timing of which was related to the migration distance (Sawyer et al., 2016). This is broadly consistent with our estimated onset of spring migration (mid‐April), but in conflict with our estimated onset of fall migration (late September). We suggest part of the discrepancy is related to our definition of the timing of changes in movement patterns as the maximum of the first derivative. We note that the timing of the local maxima of the seasonal probabilities of long‐distance movement in our work rather than the maximum of the first derivative of the seasonal trend is more consistent (and acknowledge the maximum of the first derivative, or point at which transition probabilities are increasing fastest, likely has a different biological interpretation). We stress that a long‐distance movement state is not directly analogous to a migratory movement. Instead, we speculate that our long‐distance movement state for mule deer is the conflation of two potentially distinct behaviors in the fall: long‐distance migration between seasonal ranges, and simple longer‐distance movements that precede migration or the longer‐distance movements of resident animals. Further investigation using more than two states and/or additional time‐ or space‐specific covariates is warranted to try to tease out this behavior during the fall. Recent work has revealed a diversity of migratory movement patterns for mule deer and highlighted the challenges for effective management caused by the oversimplification of migratory movements using classification strategies (van de Kerk et al., 2021). Hidden Markov movement models can ameliorate this problem by examining the daily drivers of movement states (defined by step lengths and turning angles) using additional covariates without relying on an a priori set of movement classes, thereby providing a more expansive look at the responses of mule deer to important drivers such as weather, hunting pressure, forage quality and exposure to development (Lendrum et al., 2012; Monteith et al., 2011; Rodgers et al., 2021).

The seasonal movement patterns of pronghorn are less well understood than those of mule deer. Although prior work on pronghorn in other areas has identified a diversity of migratory behaviors consistent with the resident–migrant spectrum (Jakes et al., 2018), pronghorn also tend to have larger seasonal ranges, less consistency in migrations, and a propensity to make facultative movements off seasonal ranges in (Jakes et al., 2018; Milligan et al., 2023). These movements are likely an adaptive response to their susceptibility to exposure to environmental extremes owing to their body proportions similar to other smaller‐bodied ungulates (Jakes et al., 2018; Kolar et al., 2011; Sawyer et al., 2005). We found a distinct seasonal pattern in transition probabilities of pronghorn: a seasonal increase in the probability of pronghorn to transition to a long‐distance movement state in the late fall, a longer period in which much of the population was in a long‐distance movement state, and a much larger probability of remaining in a long‐distance movement state during the winter months. We interpreted this overall pattern to reflect variability in migration timing and duration for pronghorn, as well as the potential sensitivity of pronghorn to winter weather events, and we speculate this tendency to maintain long‐distance movements over the winter (facultative winter migrations) reflects overall avoidance of areas with environmental extremes (e.g., temperature, wind, or deep snow) and increase survival. This variability in movement patterns renders the classification of pronghorn movements into discrete classes challenging, and oversimplification of complex behaviors may obscure management approaches (van de Kerk et al., 2021). These difficulties scale up to assessing drivers of variation in movement timing. Pronghorn movements are sensitive to landscape‐level disturbances such as exposure to development, fencing and land‐use practices, factors that potentially alter movement behaviors and render classification challenging (Gates et al., 2012; Milligan et al., 2021, 2023; Poor et al., 2012). Hidden Markov movement models can directly estimate the influence of these temporally and spatially finer‐scale potential drivers of variation in movement in a probabilistic framework without relying on a priori definitions of movement classes by incorporating covariates into the modeling framework. We suggest such an approach can improve our understanding of finer‐scale pronghorn movement behaviors in response to a landscape‐level disturbances while retaining the broader seasonal view of trends in movement patterns. Furthermore, we speculate that future work may assess the demographic consequences of variations in movement patterns for pronghorn by assessing variation in survival and/or reproduction associated with movements during particular times of the year, for example, a correlation between time spent in a long‐distance movement state during the winter and annual survival.

Despite some methodological limitations in our methods for inferring fine‐scale ungulate movement behavior, our work provides novel results on the seasonal movement patterns of pronghorn and mule deer. We recommend a variety of future modifications of our methods to build upon these results and improve our understanding of seasonal movement patterns. Our approach used a population‐level model and did not allow for among‐year variation in the seasonal trend in transition probabilities, which conflicts with prior results indicating a variety of movement strategies for this mule deer population and for pronghorn in general (Jakes et al., 2018; Sawyer et al., 2014, 2016). Moreover, we did not assess the potential contribution from among‐individual heterogeneity in movement patterns to the overall population‐level average (a model structure that would induce considerable technical challenges to implementation). Rather, our structure was chosen for convenience to represent an average movement model among years and individuals, which can be a valuable resource for conservation planning (Mueller et al., 2011). Similarly, we focused on seasonal movements estimated using step lengths and turning angles between successive daily locations, ignoring the finer‐scale temporal resolution that were available from the collar information and some work suggesting diel variation in behavioral states of ungulates (Beumer et al., 2020; Franke et al., 2004). Hierarchical HMMs can provide a solution by simultaneously making inference at multiple temporal scales (e.g., foraging and resting behaviors within a day, and resident and migrant behaviors among days), and accounting for among‐individual variation in movement patterns (Leos‐Barajas et al., 2017). Practically, this would allow the assessment of sources of variation in nested movement behaviors, while simultaneously assessing the diversity of those movement behaviors among individuals and/or populations.

The conservation of ungulate populations is a high priority for management agencies. Worldwide, ungulates serve important ecological and social roles (Apollonio et al., 2017), and some populations face a myriad of anthropogenic disturbances and impacts from long‐term climate change (Aikens et al., 2020; Apollonio et al., 2017; Kauffman et al., 2021; Middleton et al., 2013). Understanding ungulate movement ecology can play a key role in identifying critical habitats, movement corridors, and assessing movement responses to disturbances, thereby helping define management goals to aid conservation. Our approach using HMMs for modeling movement data was useful for gaining an initial understanding of seasonal differences in movement‐based behavior for two distinct ungulate species, pronghorn and mule deer. We recommend that future analysis of these two species could improve our work by building upon our models to incorporate additional covariates and movement states. Further, our methods could be extended to other ungulate species to understand their movement patterns at multiple spatial and temporal scales. For example, they could estimate the impact to overall seasonal patterns of movement from short‐term, severe weather events, spatially restricted anthropogenic contact (e.g., hunting pressure), or the development of energy infrastructure.

## AUTHOR CONTRIBUTIONS

**John Terrill Paterson:** Conceptualization (equal); formal analysis (equal); methodology (equal); writing – original draft (equal); writing – review and editing (equal). **Aaron N. Johnston:** Conceptualization (equal); formal analysis (equal); funding acquisition (equal); investigation (equal); methodology (equal); project administration (equal); writing – review and editing (equal). **Anna C. Ortega:** Data curation (equal); writing – review and editing (equal). **Cody Wallace:** Data curation (equal); writing – review and editing (equal). **Matthew Kauffman:** Data curation (equal); funding acquisition (equal); project administration (equal); writing – review and editing (equal).

## CONFLICT OF INTEREST STATEMENT

We declare no conflicts of interest.

## Supporting information


Data S1

## Data Availability

Supporting data are available in Johnston et al. (2023), https://doi.org/10.5066/P9MHCNXS.
