# Supplementary material for: Hidden Markov movement models reveal diverse seasonal movement patterns in two North American ungulates
Source: Ecol Evol. 2023 Jul 20;13(7):e10282. doi: 10.1002/ece3.10282 (PMC10361361; doi:10.1002/ece3.10282)
Supplement: Supplementary file 1 — Data S1 [file ECE3-13-e10282-s001.docx]

**Supplementary Information**

Details on spatial data and processing.

For pronghorn, the approximately 2-hour fix-rate data consisted of 778,788 spatial locations from 150 individuals across 4 years (2018, 2019, 2020, and 2021) for a total of 334 animal-years of location information (Table S1, Figure S1). For mule deer, the approximately 2-hour fix-rate data consisted of 654,916 spatial locations from 89 individuals across 8 years (2014, 2015, 2016, 2017, 2018, 2019, 2020 and 2021) for a total of 267 animal-years of location information (Table S2, Figure S2).

Table S1. Summary statistics for pronghorn location data and the total number of locations and individuals in each year.

|  |  |  | Number of locations per individual | | | | Sampling rate (hours) | | | |
| --- | --- | --- | --- | --- | --- | --- | --- | --- | --- | --- |
| Year | Total number of points | Number of individuals | median | sd | min | max | median | sd | min | max |
| 2018 | 222094 | 91 | 3337 | 1295.07 | 156 | 3380 | 2 | 0.74 | 1.95 | 184.00 |
| 2019 | 288268 | 99 | 3402 | 1644.10 | 16 | 4340 | 2 | 0.37 | 0.48 | 104.00 |
| 2020 | 242672 | 104 | 2961 | 1672.79 | 48 | 4351 | 2 | 0.56 | 0.11 | 146.01 |
| 2021 | 25754 | 40 | 680 | 147.26 | 38 | 796 | 2 | 0.95 | 1.95 | 146.02 |


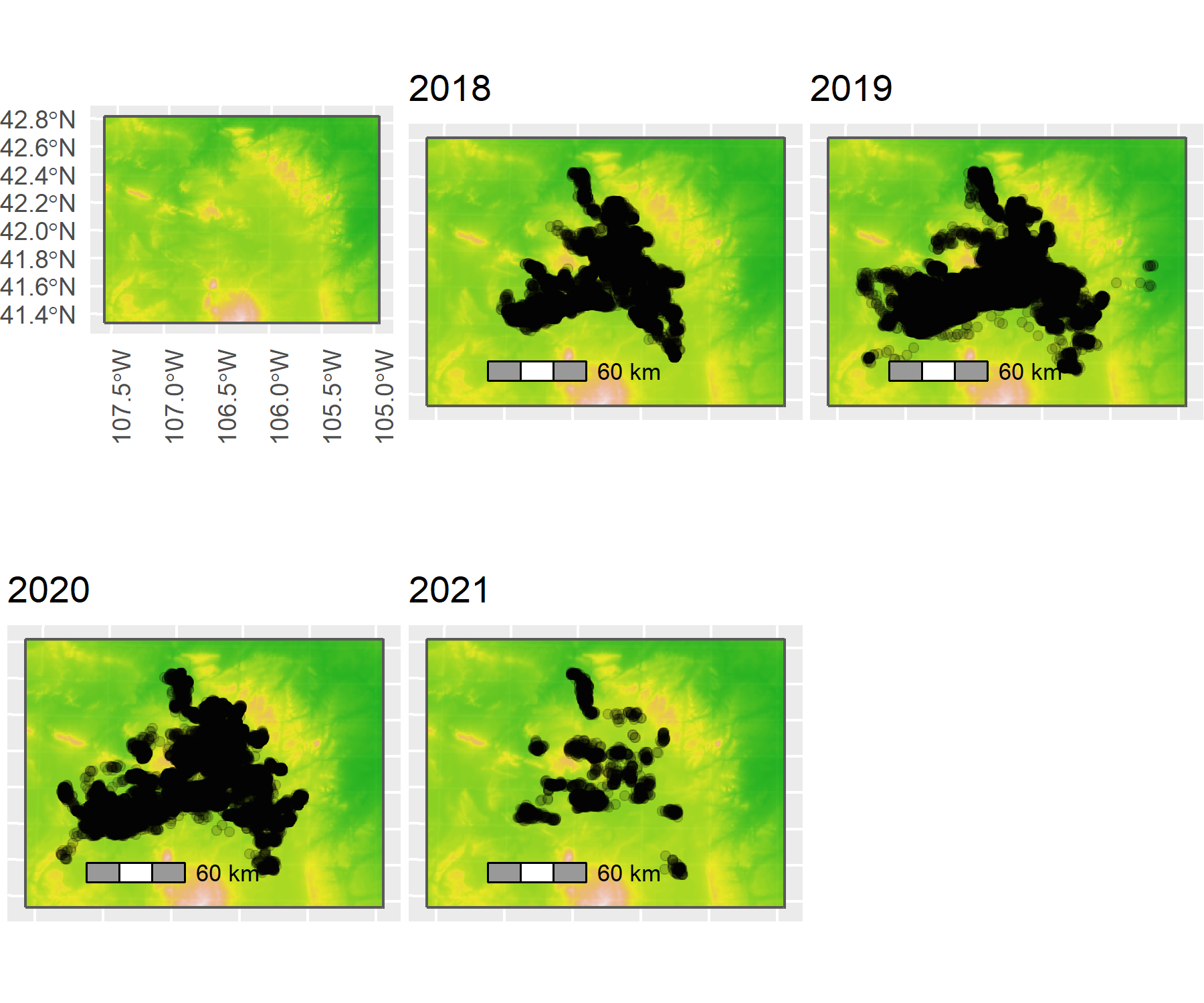


Figure S1. Raw location information for pronghorn by calendar year (each black dot is a location). The background is an elevation map that ranges from low (green: minimum elevation = 1,385 m) to high (white: maximum elevation = 3,496 m).

Table S2. Summary statistics for mule deer location data, and the total number of locations and individuals in each year.

|  |  |  | Number of locations per individual | | | | Sampling rate (hours) | | | |
| --- | --- | --- | --- | --- | --- | --- | --- | --- | --- | --- |
| Year | Total number of points | Number of individuals | median | sd | min | max | median | sd | min | max |
| 2014 | 25195 | 6 | 5111 | 2192.16 | 142 | 6159 | 1 | 14.64 | 0.12 | 1491 |
| 2015 | 2018 | 1 | 2018 | NA | 2018 | 2018 | 3 | 3.47 | 1 | 152 |
| 2016 | 48364 | 20 | 2493.5 | 907.43 | 535 | 3490 | 2 | 7.40 | 1 | 1244 |
| 2017 | 137886 | 56 | 3154 | 1341.25 | 54 | 4311 | 2 | 14.66 | 1 | 4919 |
| 2018 | 176601 | 54 | 3994 | 1424.91 | 84 | 4373 | 2 | 5.15 | 1 | 1356 |
| 2019 | 128359 | 47 | 3833 | 1693.16 | 29 | 4350 | 2 | 3.53 | 2 | 360 |
| 2020 | 104685 | 39 | 3465 | 1654.25 | 113 | 4343 | 2 | 4.49 | 1 | 336 |
| 2021 | 31808 | 44 | 934.5 | 570.50 | 64 | 2392 | 2 | 11.88 | 1 | 312 |


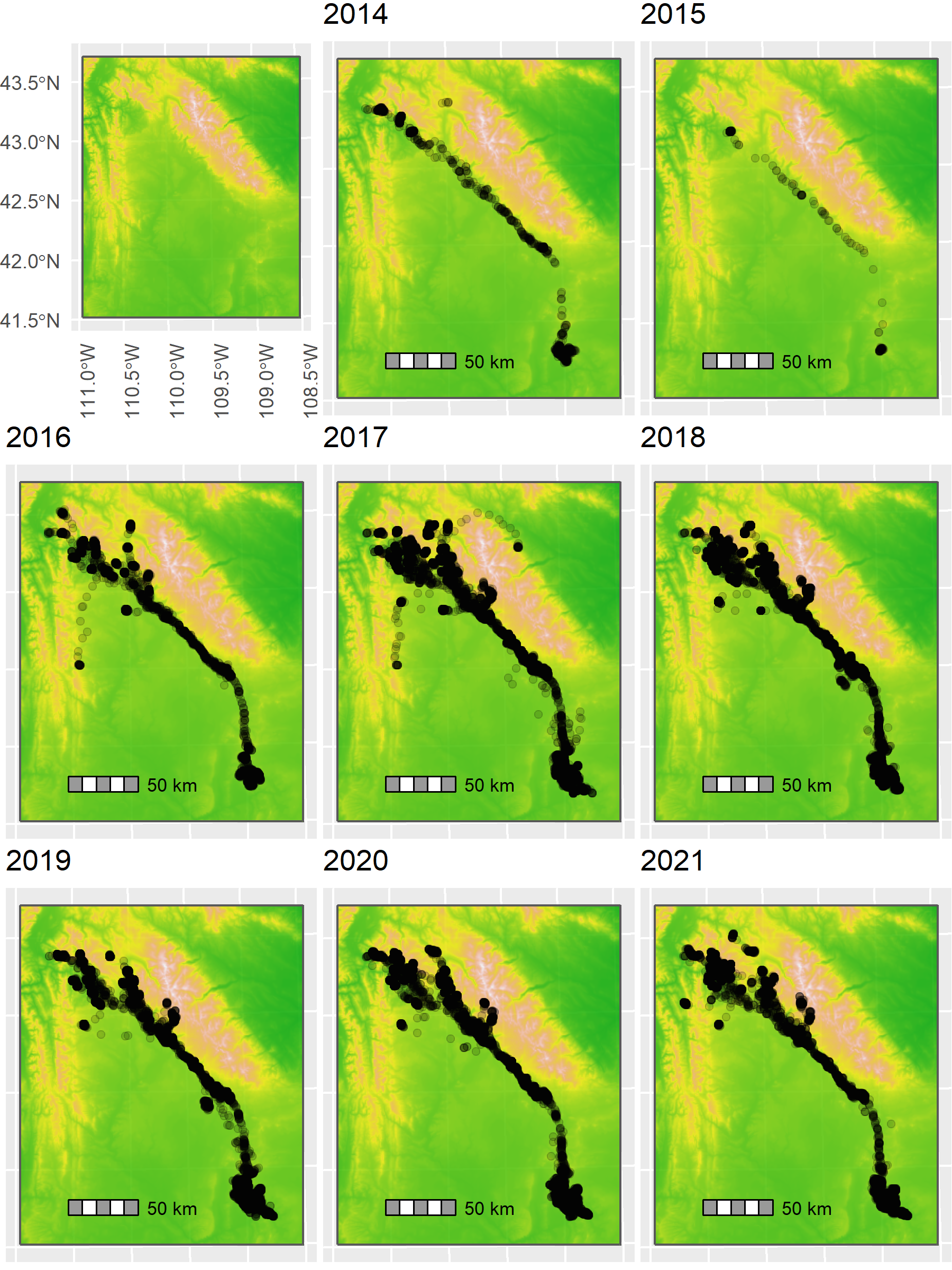


Figure S2. Raw locations for mule deer by calendar year (each black dot is a location). The background is an elevation map that ranges from low (green: minimum elevation = 1,519 m) to high (white: maximum elevation = 3,960 m).

We processed the data for analysis using the amt package (in the R programming environment) using the following steps:

1. Defined a biological year from July 1 to June 30,
2. resampled the location information to one point per day, using the point closest in time to 6 am,
3. used the make_track function to create a track object,
4. resampled the track to a lower temporal resolution of one location per day using the track_resample function with a tolerance of 8 hours to define bursts,
5. calculated the step length and turning angle between successive steps using the steps_by_burst function,
6. converted the step length from meters to kilometers, and
7. added a small constant (1E-4) to steps with a length of zero (the support of the gamma distribution is strictly positive).

The fourth step uses a function that first identifies a burst of locations with the same sampling rate prior to calculating the movement characteristics of step length and turning angle. Any gap in the time series of daily locations indicates the start of a new burst, i.e., points connected by more than one day are not used to calculate movement characteristics.

We wrote a short R function to facilitate the processing of multiple data sets:

processor = function(df){

out = df %>%

cbind(., st_coordinates(.)) %>%

st_set_geometry(NULL) %>%

rename_all(tolower) %>%

mutate(migration_year = ifelse(month(datetime) < 7, year(datetime) - 1, year(datetime)),

migration_day = floor(as.numeric(difftime(datetime, ymd_hms(paste0(migration_year, "-07-01 0:00:00")), units = "days"))) + 1) %>%

group_by(aid, migration_year) %>%

nest(data = c(id, x, y, datetime, migration_day)) %>%

mutate(sample_1 = map(

data,

function(x){

x %>%

ungroup() %>%

group_by(migration_day) %>%

mutate(diff = abs(hour(datetime) - 6)) %>%

filter(diff == min(diff)) %>%

ungroup() %>%

make_track(.x = x, .y = y, .t = datetime, all_cols = TRUE) %>%

track_resample(rate = days(1), tolerance = hours(8)) %>%

steps_by_burst(keep_cols = "end")

}

))

daily = out %>%

dplyr::select(aid, migration_year, sample_1) %>%

unnest(cols = c(sample_1)) %>%

group_by(aid, migration_year) %>%

mutate(id = cur_group_id()) %>%

mutate(sl_ = sl_ / 1000) %>%

dplyr::select(aid, steps = sl_, angles = ta_, migration_day, migration_year, x_ = x2_, y_ = y2_, id) %>%

ungroup() %>%

arrange(id) %>%

mutate(steps = ifelse(steps < 1E-4, 1E-4, steps))

return(daily)

}

After processing, our data set consisted of 66,401 daily locations of mule deer from 89 individuals across 8 years (2014 to 2021), resulting in 317 animal-years of location information, and 65,801 daily locations of pronghorn from 150 animals across 4 years (2018 to 2021), resulting in 345 animal-years of location information. Across the years, the number of animals with daily locations varied as a function of collaring efforts, mortalities and collar fixes (Figure S3). The raw data (median step length and median turning angle on each day across all individuals and years) strongly suggest some seasonality in movement patterns, as well as differences in movement timing and strategy between pronghorn and mule deer (Figure S4).


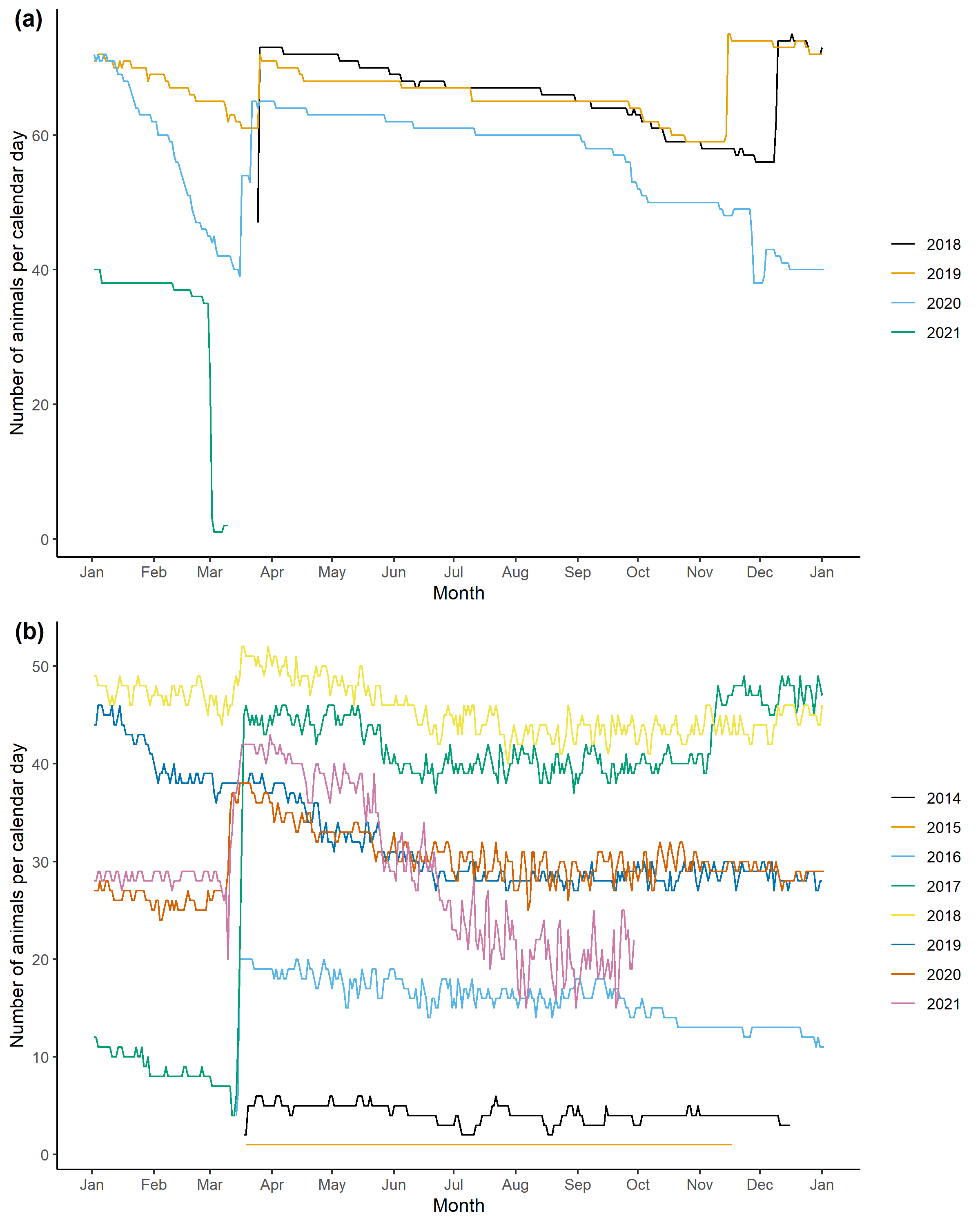


Figure S3. The number of animals with a daily location per calendar year (i.e., number of daily locations) for pronghorn (panel a) and mule deer (panel b). The number fluctuated through time based on collaring efforts, mortalities and successful collar fixes.

**
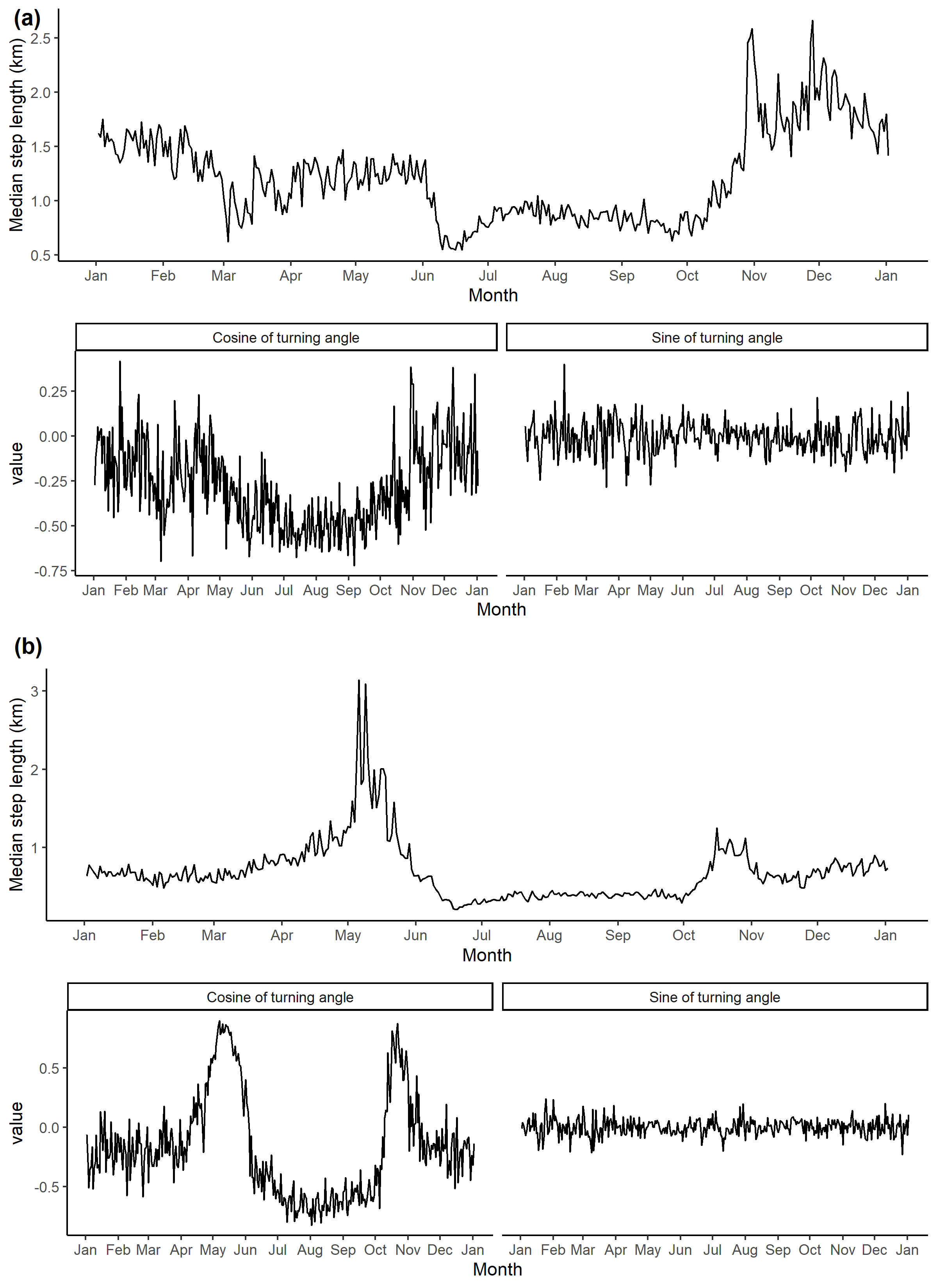
**

Figure S4. Raw movement characteristics of pronghorn (panel a) and mule deer (panel b). The top plot in each panel shows the median step length across all animals and years on each calendar day; the bottom plots show the median cosine and sine of the turning angle across all animals and years on each calendar day (a turning angle of 0 radians, i.e., straight-ahead, has a cosine of 1 and a sine of 0; a turning angle of pi radians, i.e., turning-around, has a cosine of -1 and a sine of 0).

**Supplementary Information: NIMBLE model code**

Detailed model statement and NIMBLE code (de Valpine et al., 2022).

Prior to model estimation, we constructed a spline basis using the approach of Crainiceanu et al. (20050. We first defined the covariate of interest as the number days within a biological year:

covariate = 1:365,

and calculated the spline basis for a predetermined number of knots (5 in our case) as:

knots = quantile(covariate, seq(0,1,length=(num.knots+2))[-c(1,(num.knots+2))], na.rm = TRUE)

Z_K = (abs(outer(covariate,knots,"-")))^3

OMEGA_all = (abs(outer(knots,knots,"-")))^3

svd.OMEGA_all = svd(OMEGA_all)

sqrt.OMEGA_all = t(svd.OMEGA_all$v %*% (t(svd.OMEGA_all$u)*sqrt(svd.OMEGA_all$d)))

Z = t(solve(sqrt.OMEGA_all,t(Z_K)))

For our step-length distributions we set priors on the mean and variance of the gamma distributions rather than the shape and the rate, which helped improve convergence of the MCMC chains in the Bayesian estimation algorithms. First, we defined the means of the gamma distributions as:

$\mu_{1}^{gamma}\sim Truncated Normal\left( 0, \sigma=10 \right)$,

$\delta\sim Truncated Normal\left( 0, \sigma=10 \right)$,

$\mu_{2}^{gamma}= \mu_{1}^{gamma}+ \delta$,

and the variances as:

$\sigma_{1}, \sigma_{2} \sim Truncated Normal\left( 0, \sigma=10 \right)$,

before calculating the state-specific (*i* = 1, 2) shape and rate parameters used for the sampling statement in the nimble language:

$\mathrm{shape}_{i}= \frac{\mu_{i}^{gamma}*\mu_{i}^{gamma}}{\sigma_{i}^{gamma}* \sigma_{i}^{gamma}}$, and

$\mathrm{rate}_{i}= \frac{\mu_{i}^{gamma}}{\sigma_{i}^{gamma}\sigma_{i}^{gamma}}$.

For the wrapped Cauchy distributions of turning angles, we used diffuse distributions for the state-specific (*i* = 1, 2) parameters (mean = $\mu_{i}$: concentration = $\kappa_{i}$):

$\mu_{i}\sim\mathrm{Uniform}\left( -\pi,\pi\right)$,

$\kappa_{i} \sim\mathrm{Uniform}$(0, 1).

There is no wrapped Cauchy distribution available in the nimble programming language, so we used the ones trick to add the likelihood of the turning angle using the probability density function of the wrapped Cauchy to calculate the likelihood of the observed turning angle, e.g., for an individual in state *i* the likelihood of an observed turning angle was:

$1 \sim\mathrm{Bernoulli}\left( p \right)$,

$p= \frac{1}{2\pi}* \frac{1- {\kappa_{i}}^{2}}{1+ {\kappa_{i}}^{2}-2\kappa_{i}\left( cos\left( turning angle \right) - \mu_{i} \right)}$ / C,

where C was a large enough constant to make sure $p<1$.

For the regression coefficients of the transition probability matrix model, we defined overall intercepts on the logit scale as:

($\beta_{1,2}^{0}, \beta_{2,1}^{0} \sim\mathrm{Normal}\left( 0, \sigma=2 \right)$),

and the state (*i* = 1, 2) and random effects corresponding to the regression coefficients for the spline basis as:

$b_{1}^{k} \sim\mathrm{Normal}\left( 0, \sigma_{1} \right)$ and $\sigma_{1} \sim Truncated Normal\left( 0, \sigma=10 \right)$,

$b_{2}^{k} \sim\mathrm{Normal}\left( 0, \sigma_{2} \right)$ and $\sigma_{2} \sim Truncated Normal\left( 0, \sigma=10 \right)$.

Therefore, our model for the year-specific transition probabilities on day *t* ($\gamma_{1,2}^{t,y}$, $\gamma_{2,2}^{t,y}$) was:

$$\mathrm{logit}\left( \gamma_{1,2}^{t} \right)= \beta_{1,2}^{0}+b_{1}^{1}*Z_{t, 1}+b_{1}^{2}*Z_{t, 2}+b_{1}^{3}*Z_{t, 3}+b_{1}^{4}*Z_{t, 4}+b_{1}^{5}*Z_{t, 5}$$

$$\mathrm{logit}\left( \gamma_{2,1}^{t} \right)= \beta_{2,1}^{0}+b_{2}^{1}*Z_{t, 1}+b_{2}^{2}*Z_{t, 2}+b_{2}^{3}*Z_{t, 3}+b_{2}^{4}*Z_{t, 4}+b_{2}^{5}*Z_{t, 5}$$

The following is the nimble model statement for our HMMM.

string = nimbleCode({

## priors

mu[1] ~ T(dnorm(0, 10), 0, )

eps ~ T(dnorm(0, 10), 1, )

mu[2] <- mu[1] + eps

sigma[1] ~ T(dnorm(0, 10), 0, )

sigma[2] ~ T(dnorm(0, 10), 0, )

kappa[1] ~ dunif(0, 1)

kappa[2] ~ dunif(0, 1)

loc[1] ~ dunif(-pi, pi)

loc[2] ~ dunif(-pi, pi)

# transform mean and SD to shape and rate

for(n in 1:2)

shape[n] <- mu[n]*mu[n]/(sigma[n]*sigma[n])

for(n in 1:2)

rate[n] <- mu[n]/(sigma[n]*sigma[n])

## set up transition probabilities

for (k in 1:5){

b[k] ~ dnorm(0, sd = sigmab1)

b2[k] ~ dnorm(0, sd = sigmab2)

}

sigmab1 ~ dunif(0, 10)

sigmab2 ~ dunif(0, 10)

int1 ~ dnorm(0, sd = 2)

int2 ~ dnorm(0, sd = 2)

for(t in 2:T) {

prob[t, 1, 1] <- 1 - prob[t, 1, 2]

prob[t, 1, 2] <- ilogit(int1 +

b[1]*Z[t,1]+b[2]*Z[t,2]+b[3]*Z[t,3]+b[4]*Z[t,4]+b[5]*Z[t,5])# +

# b[6]*Z[t,6]+b[7]*Z[t,7]+b[8]*Z[t,8]+b[9]*Z[t,9]+b[10]*Z[t,10])

prob[t, 2, 1] <- ilogit(int2 +

b2[1]*Z[t,1]+b2[2]*Z[t,2]+b2[3]*Z[t,3]+b2[4]*Z[t,4]+b2[5]*Z[t,5])# +

# b2[6]*Z[t,6]+b2[7]*Z[t,7]+b2[8]*Z[t,8]+b2[9]*Z[t,9]+b2[10]*Z[t,10])

prob[t, 2, 2] <- 1 - prob[t, 2, 1]

}

for (i in 1:n.numbers){

phi[i,1:2] ~ ddirch(init[1:2])

}

# likelihood computation

for (i in 1:N) {

idx[i,first[i] - 1] ~ dcat(phi[index[i], 1:2])

for (t in first[i]:last[i]){

angles[i,t] ~ dunif(-pi, pi)

# likelihood for steps

steps[i,t] ~ dgamma(shape = shape[idx[i, t]], rate = rate[idx[i, t]]) # a is the shape parameter

idx[i, t] ~ dcat(prob[times[i, t], idx[i, t-1], 1:2])

# likelihood for angles

ones[i,t] ~ dbern((1/(2*pi)*(1-pow(kappa[idx[i, t]],2))/(1+pow(kappa[idx[i, t]],2)-2*kappa[idx[i, t]]*cos(angles[i,t]-loc[idx[i, t]])))/10)

}

}

pi <- 3.141593

})

Crainiceanu, C., Ruppert, D., & Wand, M. P. (2005). *Bayesian analysis for penalized spline regression using WinBUGS*.

de Valpine, P., Paciorek, C., Turek, D., Michaud, N., Anderson-Bergman, C., Obermeyer, F., Cortes, C. W., Rodrìguez, A., Lang, D. T., & Paganin, S. (2022). *NIMBLE: MCMC, Particle Filtering, and Programmable Hierarchical Modeling* (0.12.2) [Computer software]. https://doi.org/10.5281/zenodo.1211190
